# Clinical testing of *BRCA1* and *BRCA2*: a worldwide snapshot of technological practices

**DOI:** 10.1038/s41525-018-0046-7

**Published:** 2018-02-15

**Authors:** Amanda Ewart Toland, Andrea Forman, Fergus J. Couch, Julie O. Culver, Diana M. Eccles, William D. Foulkes, Frans B. L. Hogervorst, Claude Houdayer, Ephrat Levy-Lahad, Alvaro N. Monteiro, Susan L. Neuhausen, Sharon E. Plon, Shyam K. Sharan, Amanda B. Spurdle, Csilla Szabo, Lawrence C. Brody, on behalf of the BIC Steering Committee

**Affiliations:** 1 Departments of Cancer Biology and Genetics and Internal Medicine, Comprehensive Cancer Center, The Ohio State University, Columbus, OH 43210 USA; 2 Fox Chase Cancer Center, Philadelphia, PA 19111 USA; 3 Department of Laboratory Medicine and Pathology, Mayo Clinic, Rochester, MN 55905 USA; 4 USC Norris Comprehensive Cancer Center, University of Southern California, Los Angeles, CA 90033 USA; 5 Faculty of Medicine, University of Southampton, Southampton, S016 5YA UK; 6 Departments of Human Genetics, Medicine and Oncology, McGill University, Montreal, QC Canada H4A 3J1; 7 Family Cancer Clinic, Netherlands Cancer Institute, Amsterdam, 1006 BE Netherlands; 8 Oncogenetics and INSERM U830, Institut Curie, Paris and Paris Descartes University, Paris, 75248 France; 9 Faculty of Medicine, Shaare Zedek Medical Center, Hebrew University of Jerusalem and Medical Genetics Institute, Jerusalem, 9103102 Israel; 10 Department of Cancer Epidemiology, Moffitt Cancer Center, Tampa, FL 33612 USA; 11 Department of Population Sciences, Beckman Research Institute of City of Hope, Duarte, CA 91010 USA; 12 Baylor College of Medicine, Houston, TX 77030 USA; 13 Mouse Cancer Genetics Program, Center for Cancer Biology, National Cancer Institute, National Institutes of Health, Frederick, MD 21702-1201 USA; 14 Genetics and Computational Biology Division, QIMR Berghofer Medical Research Institute, Herston, Brisbane, QLD QLD 4006 Australia; 15 National Human Genome Research Institute, National Institutes of Health, Bethesda, MD 20892 USA

## Abstract

Clinical testing of *BRCA1* and *BRCA2* began over 20 years ago. With the expiration and overturning of the *BRCA* patents, limitations on which laboratories could offer commercial testing were lifted. These legal changes occurred approximately the same time as the widespread adoption of massively parallel sequencing (MPS) technologies. Little is known about how these changes impacted laboratory practices for detecting genetic alterations in hereditary breast and ovarian cancer genes. Therefore, we sought to examine current laboratory genetic testing practices for *BRCA1*/*BRCA2*. We employed an online survey of 65 questions covering four areas: laboratory characteristics, details on technological methods, variant classification, and client-support information. Eight United States (US) laboratories and 78 non-US laboratories completed the survey. Most laboratories (93%; 80/86) used MPS platforms to identify variants. Laboratories differed widely on: (1) technologies used for large rearrangement detection; (2) criteria for minimum read depths; (3) non-coding regions sequenced; (4) variant classification criteria and approaches; (5) testing volume ranging from 2 to 2.5 × 10^5^ tests annually; and (6) deposition of variants into public databases. These data may be useful for national and international agencies to set recommendations for quality standards for *BRCA1/BRCA2* clinical testing. These standards could also be applied to testing of other disease genes.

## Breast cancer: Labs differ widely in BRCA testing protocols

Global protocols and standards differ widely when it comes to analyzing variants in the *BRCA1* and *BRCA2* cancer susceptibility genes. A team led by Amanda Toland from the Ohio State University Comprehensive Cancer Center in Columbus, Ohio, USA, surveyed 86 laboratories around the world about their testing practices for *BRCA1/2*, genes linked to hereditary cases of breast and ovarian cancer. The vast majority of responding labs—93%—used next-generation sequencing technologies; only 6 relied on traditional Sanger sequencing methods. But otherwise, the researchers found, laboratories differed widely in their approach to analyzing *BRCA*—including in the extent of variant confirmation, whether non-coding DNA regions were sequenced, and the techniques used to detect large genomic rearrangements. The survey results now provide a baseline dataset for organizations to formulate global best-practice guidelines for *BRCA* testing.

## Introduction

Clinical genetic testing of *BRCA1* and *BRCA2* began in the mid-1990s, but was mainly limited to one laboratory in the United States (US) and a small number of laboratories in Australia and Europe. Twenty-five years later the number and types of patients being offered *BRCA1/BRCA2* testing has changed dramatically due in part to changes in patent laws and increased recognition of potential benefits of testing. Additionally, advances in high-throughput sequencing technology have enabled laboratories to offer tests that cover more genes. The testing panels are less expensive than older tests and feature shorter turn-around times (TAT). These changes in access and technology have led to a similarly intense increase over the last 10 years in the number of laboratories offering clinical genetic testing of *BRCA1*/*BRCA2* specifically, multi-gene cancer gene panels for germline and somatic variant analysis, as well as companies that offer whole-exome and whole-genome studies that capture mutation information on these genes. This has led to a diverse array of options from which cancer genetics care providers can choose.

In 2013, a survey of 13 US laboratories offering *BRCA1/BRCA2* sequence analysis revealed a large number of differences in technology, gene coverage, analytic sensitivities, ability to detect large rearrangements, cost, single-site analyses, and frequency of reporting variant of uncertain significance (VUS).^1^ Since 2013, many additional laboratories now offer *BRCA1/BRCA2* testing, including laboratories that perform somatic mutation analyses. Given the expanded testing and challenges faced by clinicians and patients interested in comparing test offerings, the Breast Cancer Information Core (BIC) Steering Committee developed and administered a survey aimed to address the question of variation in clinical laboratory practices for *BRCA1/BRCA2* testing around the world. The BIC is an open access, online database, which began in 1995, to catalog sequence variants in *BRCA1/BRCA2*.^2^ The goal of this study was to obtain a snapshot of current genetic testing laboratory practices for *BRCA1*/*BRCA2* worldwide. We believe this study highlights similarities and differences between laboratories including technologies utilized, regions of the genes commonly assessed, as well as other quality control metrics employed. These findings help us to understand international differences in testing protocols and standards.

## Results

### Laboratory locations

**Fig. 1:**
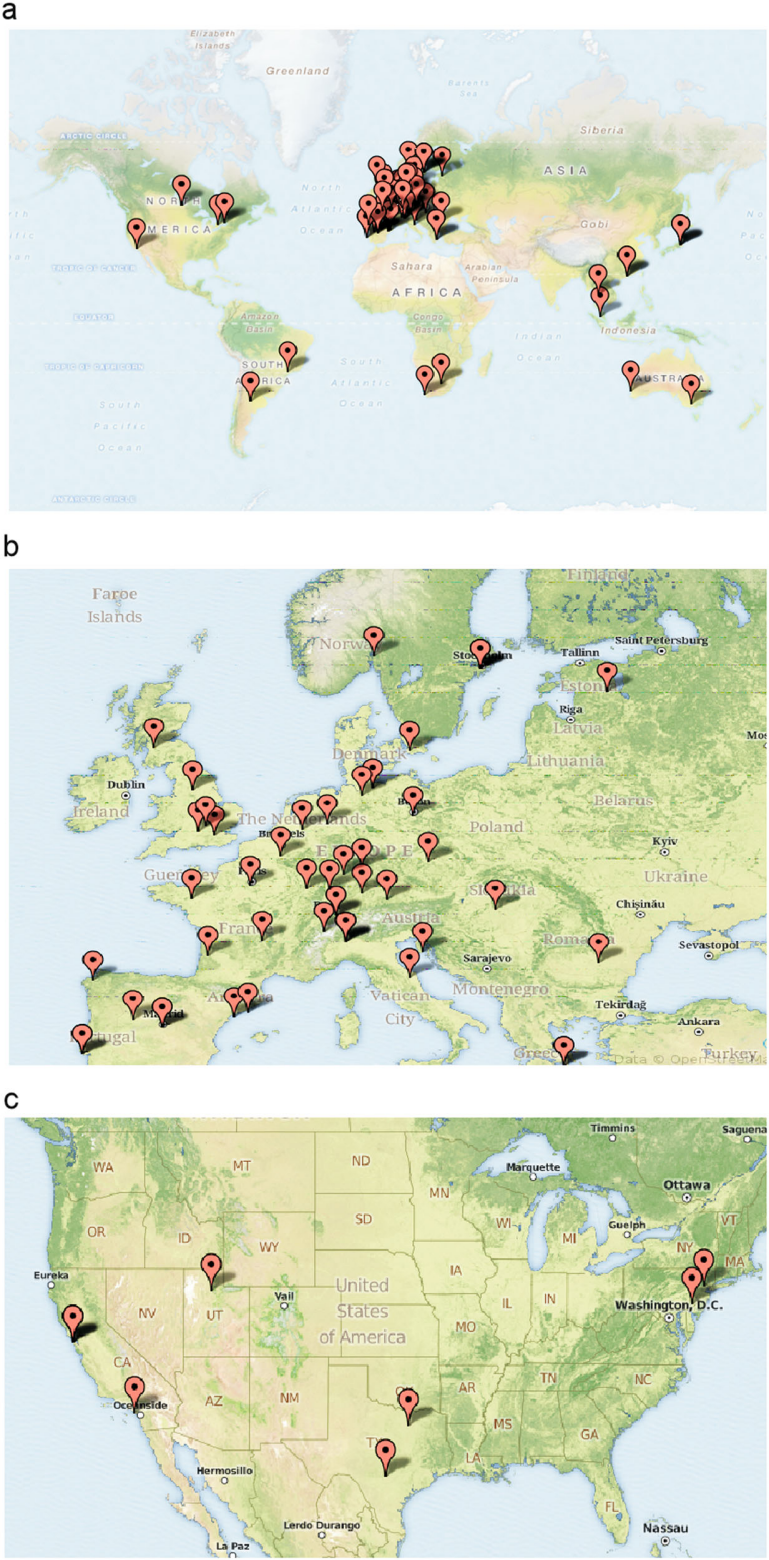
Geographical location of participating *BRCA1/BRCA2* testing laboratories. The geographical location of participating laboratories is shown as pins on the world map for non-US laboratories (**a**), European laboratories (**b**), and US laboratories (**c**). Only laboratories that completed at least half of the survey questions are shown. Two of the US laboratories have overlapping pins as they are located in the San Francisco Bay Area. OpenStreetMap and ZeeMaps hold the copyright for the maps

Survey links were sent to *BRCA1/BRCA2* genetic testing laboratories around the world. Every continent, except Antarctica, was represented by at least one testing laboratory (Fig. 1a). The bulk of testing laboratories responding to the survey were from Europe which reflects the demographics of the laboratories which were sent the survey invitation (Fig. 1b). Eight US laboratories completed the survey (Fig. 1c).

### Technologies used

**Fig. 2:**
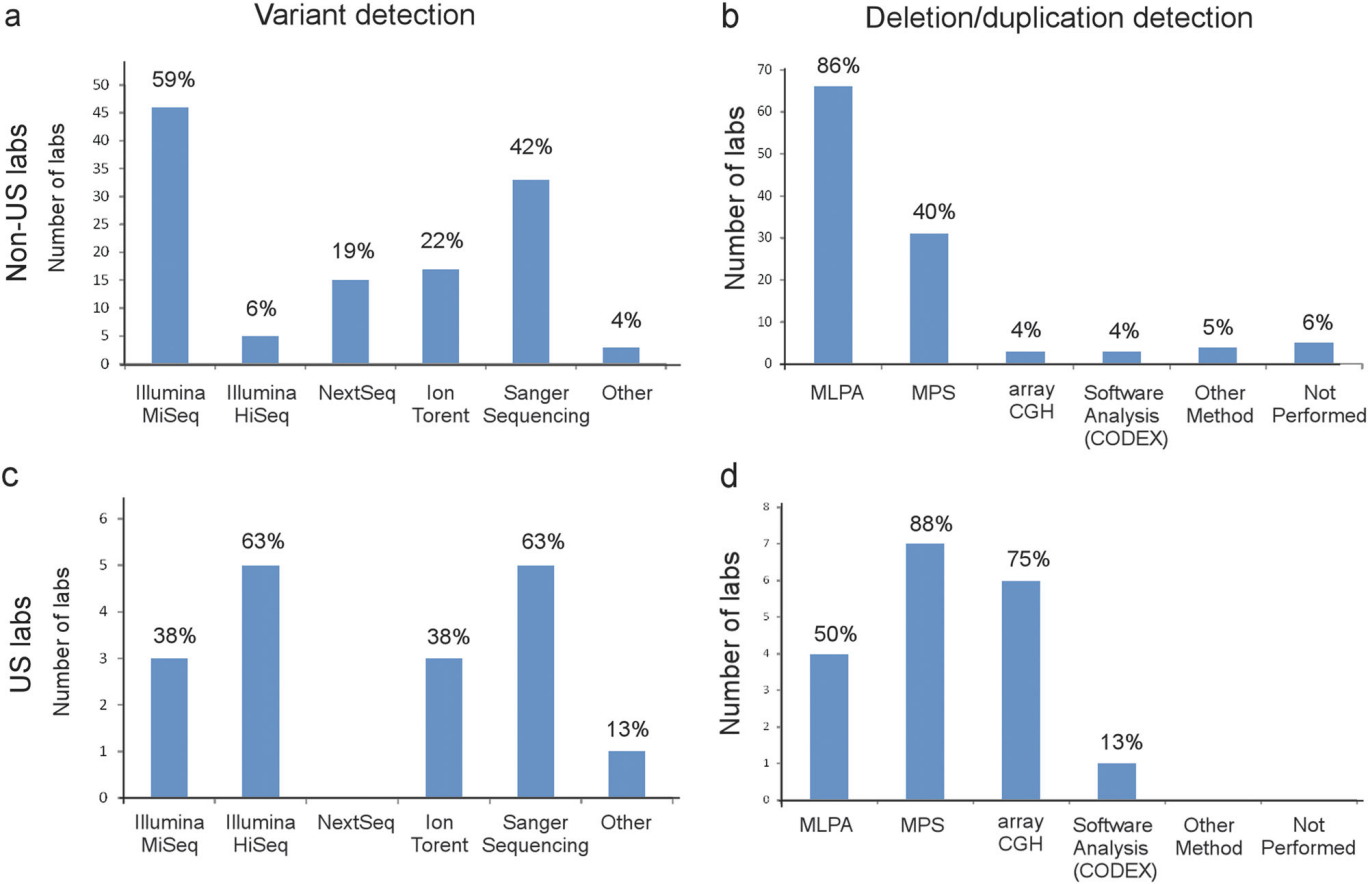
Methods used for *BRCA1/BRCA2* variant identification. The number and percentage of non-US (**a/b**) and US laboratories (**c/d**) reporting use of each method for identification of *BRCA1/BRCA2* sequence variants (**a/c**) and large rearrangements (**b/c**) are noted. The percentage of laboratories reporting the use of more than one technology for variant detection was **a** 40%, **b** 38%, **c** 75%, and **d** 75%. Multiplex ligation-dependent probe amplification (MPLA); massively parallel sequencing (MPS); array comparative genomic hybridization (array CGH)

Massively parallel sequencing (MPS) was the most common method utilized by non-US laboratories to identify *BRCA1/BRCA2* variants with the most common platforms being Illumina MiSeq (59%; 46 of 78 laboratories) and Ion Torrent (22%, 17 of 78 laboratories) (Fig. 2a). Of the 78 non-US laboratories responding, six reported Sanger sequencing as their only method of sequencing (8%) and 27 additional laboratories (35%) used Sanger as at least one modality, mostly to confirm variants identified by other methods. Twenty-six of 33 (79%) non-US laboratories reporting on both technology used and use of gene-panels include ten or more genes on their panel; seven laboratories reported using MPS for *BRCA1/2* only. Many laboratories (31/78; 40%) reported using multiple technologies sequentially for initial discovery. There was less variability in the approach for large rearrangement analysis with 86% of non-US laboratories (66 of 77) utilizing multiplex ligation-dependent probe amplification (MLPA); however, 31% (24 of 77) also used data from a MPS platform, some noting that they used MLPA analyses to confirm rearrangements identified by MPS (Fig. 2b). Five of 77 laboratories (6%) did no duplication/deletion analyses, and one reported sending DNA to an external laboratory if no variants were detected by MPS. All eight US-laboratories used MPS technologies as one modality for sequence variant detection, and six reported using more than one technology (Fig. 2c). All US-laboratories responding offer gene panels and have panels available that include more than ten genes. Five of the eight US-laboratories also reported using Sanger sequencing, mostly for confirmation of variants found through other methods. Only half of the US laboratories used MLPA which differs from the non-US laboratories (66 of 77; 86%) (Fig. 2d). Seven of the US laboratories reported using more than one platform to identify large sequence variants; six used chromosomal microarray analysis or array comparative genomic hybridization (aCGH) (75%) in combination with MPS (five of eight; 63%). One US laboratory reported solely using MPS for detection of large sequence events.

The survey did not specifically ask testing laboratories whether they only performed panel analyses, only if they performed single gene analyses or did both types of tests. However, laboratories indirectly answered this question when addressing TAT. All eight US laboratories responding to the survey offered panel-testing of multiple cancer susceptibility genes. Of the eight, only one does not offer single gene analysis. For the 47 non-US laboratories, 40 (85%) noted that they perform multi-gene panels; two did not respond to the *BRCA1/BRCA2* only TAT question. Forty-five laboratories reported TAT for *BRCA1/BRCA2* testing of which eight do not perform panel testing (18%).

### Regions of *BRCA1*/*BRCA2* sequenced

**Fig. 3:**
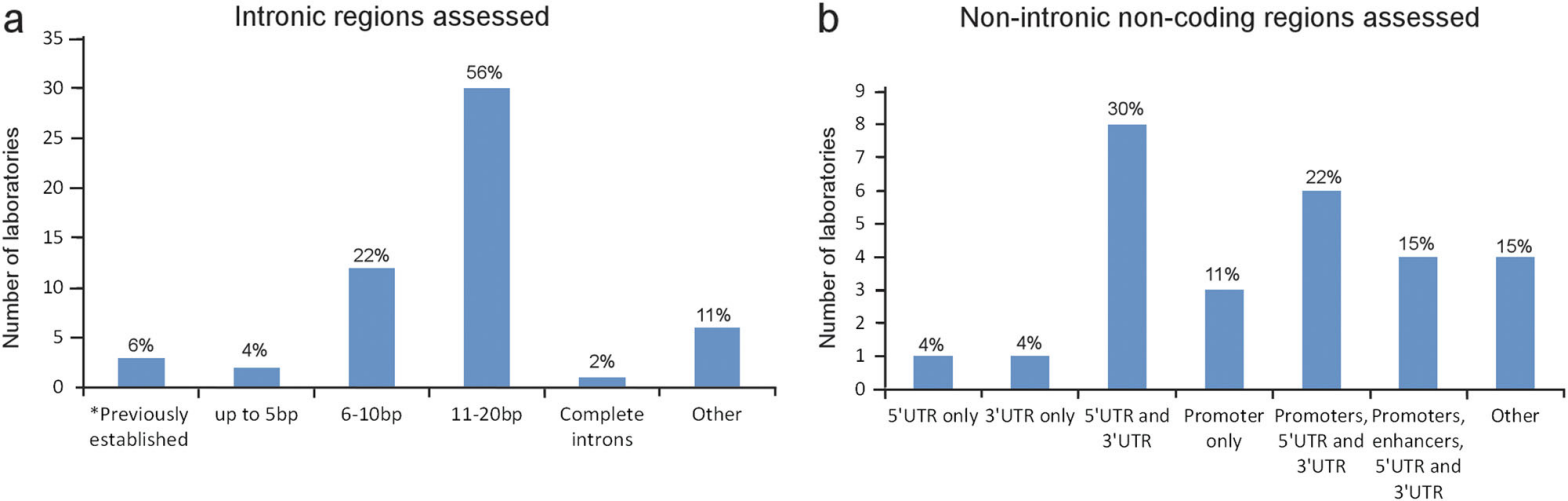
Non-coding regions assessed. Of 54 non-US laboratories reporting, the number that sequenced intronic regions (**a**) or non-coding non-intronic regions (**b**) are shown for different categories. Most of the intronic sequence refers to sequence near intron/exon boundaries. *Only sequencing previously established clinical relevant intronic variants. Other for introns includes multiple categories of size of introns and other for non-intronic regions includes “non-specified”, partial or only intronic non-coding regions. Twenty-seven laboratories answered the non-intronic regulatory regions of *BRCA1*/*BRCA2* question

For *BRCA1*/*BRCA2* analyses, coding exons were covered in full by all laboratories but only six of 70 non-US laboratories total (9%) and no US laboratories reported doing full intronic sequencing. Of the 54 non-US laboratories reporting the length of intronic sequence included in their tests, 30 (56%) sequenced 11–20 bp of intronic sequence, 12 (22%) sequenced 6–10 bp of intronic sequence and two laboratories sequenced up to 5 bp at intron/exon junctions (Fig. 3a). Twelve non-US laboratories responded with other answers including four laboratories that performed complete intronic sequencing. Twenty-three of 54 non-US laboratories (43%) sequenced non-intronic non-coding regions of *BRCA1*/*BRCA2* including promoters, enhancers, 3′untranslated regions (UTRs), and 5′UTRs (Fig. 3b). US laboratories sequenced similar intronic regions as non-US laboratories with three sequencing 11–20 bp (43%), one sequencing 6–10 bp (14%), one sequencing 20 bp proximal to the 5′ end of an exon and 10 bp distal to the 3′ end of an exon (14%), one sequencing up to 5 bp of introns plus all previously established intronic variants (14%) and one sequencing all previously established intronic variants. Six of seven US laboratories reported sequencing additional non-intronic, regulatory regions of *BRCA1*/*BRCA2*. One laboratory noted that they reported variants in established clinically relevant non-coding regions. We did not ask specifically whether the laboratories reported sequence variants from these regions.

### Variant confirmation

If a sequence variant was identified, over half of non-US (56%; 29 of 52) and US laboratories (71%, five of seven) confirmed all variants of uncertain significance (VUS), pathogenic and likely-pathogenic sequence variants by another method. Twenty-seven percent of non-US laboratories and 29% of US laboratories confirmed only pathogenic or likely pathogenic variants. Sanger sequencing was the most common method to validate variants (98%; 50 of 51 non-US laboratories and 86%, six of seven US laboratories), although 43% of US laboratories (three of seven) began confirmation by repeating analysis with the original technology. MLPA was used to confirm duplication or deletion variants in 61% of non-US laboratories (31/51) and 57% of US laboratories (3/7). Other methods used to confirm large rearrangements by US laboratories included aCGH, quantitative PCR (qPCR), and the PacBio system.

### Coverage of Sequencing

There was a large range in both average read depth and minimum read depth required to meet quality assurances for MPS (Table 1). Of the 37 non-US laboratories reporting read-depths, the median read depth across all genes on their cancer gene platforms was 476 with an average read depth of 759. These values were lower for *BRCA1/BRCA2* with a median read depth of 100 and an average read depth of 484. US laboratories reported a median read depth across all MPS platforms of 738 with a median of 425 and a higher read-depth for *BRCA1/BRCA2* (average = 820, median = 500). Forty-two non-US laboratories reported a minimum read depth required for *BRCA1/BRCA2* analysis; these ranged from 20 to 500 reads for *BRCA1/BRCA2*. When an exon failed to meet the minimum quality standards for that laboratory, most non-US laboratories used Sanger sequencing for the failed region (67%, 31 of 46) or a combination of repeating the entire or part of the assay and/or Sanger sequencing the affected region (22% 10 of 46). A small number of non-US laboratories repeated the entire assay (4%; 2 of 46) or repeated the assay around the affected region (2.1%, 1 of 46) without any Sanger sequencing. In contrast, four of the US laboratories (50%) repeated the entire assay and/or performed Sanger sequencing of the affected region.

**Table 1:** Massively parallel sequencing average and minimum read depths

	Number of reads non-US laboratories		Number of reads US laboratories	
	*BRCA1/BRCA2* specifically^a^	All genes on panel^b^	*BRCA1/BRCA2*^c^ specifically	All genes on panel^c^
Average read depth (median read depth)	483.5 (101)	758 (476)	820 (500)	738.3 (425)
Range of read depth	40–7000	40–7000	250–2400	150–2400
Average minimum read depth (median read depth)	86.6 (50)	77.7 (40)	37.8 (50)^d^	33.5 (50)^d^
Range of minimum read depth	10–500	10–500	15–50^d^	15–50^d^

^a^40 laboratories reporting

^b^39 laboratories reporting

^c^7 laboratories reporting

^d^Some laboratories reported a minimum read depth of 15 and will visually inspect reads of 15–50 before determining whether to perform Sanger sequencing analysis. Minimum refers to the minimum at which no additional studies are performed

### Analytics-sensitivity/specificity

Forty-six non-US laboratories answered questions related to the analytical sensitivity of their *BRCA1/BRCA2* testing. Of these, 16 (30%) had not calculated an analytic sensitivity for identification of known *BRCA* variants. Twenty-six non-US laboratories (52%) provided a value with an average analytical sensitivity of 98.3 and a median of 99. All eight US laboratories answered questions related to sensitivity and provided an estimated analytical sensitivity for detection of sequence variants with an average of 99.6% and a range of 98–100%. Seven of eight US laboratories reported the number of samples used to calculate the sensitivity with an average of 598, a median of 500, and a range of 20–1864.

The laboratories were also asked questions about the sensitivity, false discovery rate (FDR), and positive predictive value (PPV) of their method for identifying insertion/deletions (indels). Of the 24 non-US laboratories responding, 21% (5/24) did not know this information. Of the 16 laboratories that reported sensitivity as a value, the average was 97% and the median was 99.5% with a range of 85.2 to 100%. Four US laboratories reported a FDR with an average of 2.25%, a median of 2.5% and a range of 0–4. Multiple laboratories noted that FDR and PPV rates were difficult to determine. Of the 20 non-US laboratories responding with values, the average reliably detected indel size was 31 bp with a median of 21 bp and a range of 1–104 bp. One non-US laboratory, using Sanger sequencing, noted that they could reliably detect indels of less than 100 bp. For the five US laboratories responding, one noted that sensitivity, FDR, and PPV for indels were not calculated. For the other four laboratories, three noted a sensitivity of 100% with 95% confidence intervals of above 99.9% and a specificity of 100% and/or a FDR of 0%. Six US laboratories reported the size of reliably detected indels. Reliable detection varied between deletions and duplications; deletions of an average size of 33 bp with a median of 30 bp and a range of 20–40 bp and duplications of an average length of 23 bp, a median of 25 bp and a range of 10 to 40 bp were considered optimal for detection.

### Variant classification

A number of variant interpretation guidelines were used. All reporting laboratories responded that variant interpretation was performed by in-house staff. For non-US laboratories, the majority had a board certified medical geneticist or molecular geneticist on their interpretation team (74%, 35/47). The remaining laboratories had genetic counselors, individuals with clinical genetics expertise related to the specific gene or an expert panel performed variant classification. Almost half of non-US laboratories (47%, 22 of 47) and 38% of US laboratories (3/8) reported using American College of Medical Genetics and Genomics (ACMG) guidelines for variant interpretation.^3^ The remaining laboratories reported using in-house guidelines (non-US laboratories 28% 13 of 47; US laboratories 63%) or country or organization-specific guidelines (non-US laboratories 21%). However, in the text descriptions, many of the laboratories using “in house” guidelines relied at least in part on guidelines from expert agencies. Guidelines or reference databases used to aid in classification included Evidence-based Network for the Interpretation of Germline Mutant Alleles (ENIGMA) (enigmaconsortium.org/library/general-documents), Vereniging Klinisch Genetische Laboratoriumdiagnostiek (www.vkgl.nl/nl), Association for Clinical Genomic Science (www.acgs.uk.com), and others.^4^ Laboratories described a mixture of different resources and databases used to help in classification including ClinVar, literature searches, Alamut Visual (www.interactive-biosoftware.com/alamut-visual), Sorting Intolerant From Tolerant (SIFT)(sift.jcvi.org), Polyphen2 (genetics.bwh.harvard.edu/pph2), dbSNP (www.ncbi.nlm.nih.gov/projects/SNP), Breast Information Core Database (BIC)(research.nhgri.nih.gov/bic), Leiden Open Variation Database (LOVD)(www.lovd.nl/3.0/home), Universal Mutation Database (UMD)(www.umd.be/BRCA1), and BRCA Exchange (brcaexchange.org).^5–9^

Interpretation of VUS is an important clinical issue. Interestingly, more than half (52%, 24 of 46) of the responding non-US laboratories had not specifically calculated their *BRCA1/BRCA2* VUS rates. Of those that determined their rate of *BRCA1/BRCA2* VUS, these varied widely from 3–50% with an average and median VUS rate for *BRCA1* of 14 and 13% and for *BRCA2* of 16 and 13%. Some caution should be taken for interpretation of these figures as the definition of VUS (e.g., all VUS versus VUS per individual) and thresholds for calling vary between laboratories; also some laboratories may have reported all sequence variants and not just VUS (Supplemental Table 1). For the five US laboratories that calculated *BRCA1/BRCA2* VUS frequencies, the percentage of individuals/tests with a VUS ranged from less than 2 to 6%. Segregation analyses can be helpful for classifying variants. Seventy percent (33 of 47) of non-US laboratories offered variant-specific testing for family members when a VUS was identified and another 15% of laboratories (7 of 47) offered VUS testing depending on the circumstance. All US laboratories offered VUS-family studies, but testing was limited to defined circumstances (Supplementary Table 2).

As VUS can be reclassified as additional data become available, we asked how often laboratories reclassified VUS. Most non-US laboratories (57%, 27 of 47) specified that reclassification was done on an ad hoc basis, 9% (4 of 47) re-assessed VUS at least annually, and 23% of laboratories (11 of 47) re-assessed VUS every 1–3 years. Three non-US laboratories did not reassess variants (6%). This is in contrast to US laboratories in which 57% (four of seven) reassessed variants less than once a year, 29% (two of seven) reclassified variants on an ad hoc basis and one laboratory reassessed VUS every 1–3 years. One US laboratory reported that VUS were assessed daily due to automated reclassification tools used in real time. When a VUS was reclassified, regardless of classification, all eight US laboratories and 39% (18/46) of non-US laboratories recontacted the ordering provider. Thirty-three percent (15/46) of non-US laboratories recontacted the ordering provider only when the VUS had been reclassified as pathogenic or likely pathogenic. The remaining non-US laboratories did not automatically contact the ordering physician (11%, 5 of 46) or only did so when asked by the ordering provider (13%, 6 of 46).

To aid in classifying missense and other non-truncating variants as pathogenic or not, it is useful to know how frequently a variant has been observed in populations being tested. One third of non-US laboratories (36%, 16 of 45) did not report any variants to any databases, one third (33%, 15 of 45) shared *BRCA1/BRCA2* data with multiple open-access databases, and the remainder of labs reported to only one database (22%, 10 of 45) or to private or member-only databases (9%, 4/45). Of laboratories that reported variants the most common databases were LOVD (40%, 18 of 45), BIC (36%, 16 of 44) and ClinVar (27%, 12 of 45). US laboratories were more likely to submit sequence data to public databases. Seven of eight US laboratories (88%) reported sequence variants to ClinVar; one laboratory did not report to public or private databases.

### Testing volume

Variant interpretation, TAT, and some quality measures may be impacted by the testing volume. There was significant variation among laboratories responding to this survey question. Forty-three non-US laboratories described the number of *BRCA1/BRCA2* tests performed between October 2015 and September 2016; the number of tests ranged from 2 to 2025 with an average number per year of 568 and a median of 300. Twelve laboratories performed fewer than 100 tests per year. Only three US laboratories answered the question on the number of tests performed in the year from October 2015 to September 2016. The range was ~45,000 to 252,223 which surpassed all non-US laboratories by an order of magnitude (maximum of 2025); this may be influenced by population size of country, the length of time that testing has been available to clinicians, country-specific guidelines for testing, and differences in marketing practices.

### Client-related concerns

**Fig. 4:**
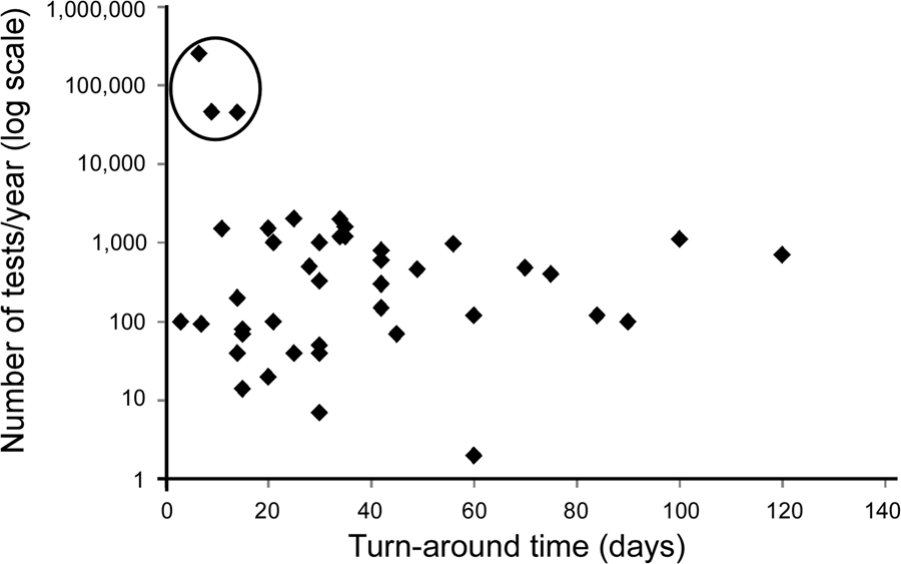
Turn-around time per testing volume. The turn-around time (TAT) in days is plotted as a function of number of *BRCA1/BRCA2* tests performed in a one-year time period for the three US laboratories and 38 non-US laboratories who reported values for both questions. US laboratories are indicated by a circle. No correlation was found between TAT and testing volume for all 41 laboratories or the 38 non-US laboratories (Pearson’s correlation = −0.23 and 0.02 respectively)

When there is a choice of laboratory and the patient and primary care provider are using *BRCA1/BRCA2* testing results to aid in surgical decision making, TAT is an important consideration. Of 38 non-US laboratories reporting, there was an average TAT for gene-panel testing of 6.5 weeks with a median of 5.6 weeks and a range of 11 days to 6 months. For *BRCA1/BRCA2* single-gene or small panel analysis specifically, there was an average TAT of 4.9 weeks (median = 4.0 weeks, range 0.5–4 months) for the 45 laboratories responding. This contrasts with US laboratories that had a much shorter average TAT of 2.4 weeks for gene-panels of over ten genes with a median of 2.6 weeks and a range of 1.1 to 3.5 weeks. For single gene analysis or small panels (six US laboratories reporting), there was an average TAT of 1.7 weeks with a median of 1.5 weeks and a range of 6.5 days to 2 weeks. One US laboratory noted that there were additional options to expedite test results if requested. To determine if TAT was influenced by the number of tests performed per year, we plotted the TAT for *BRCA1/BRCA2* tests in days by number of tests performed per year for the three US laboratories and 38 non-US laboratories for which both values were present (Fig. 4). There was no correlation between TAT and testing volume (Pearson’s correlation all labs combined = −0.28; non-US labs only was 0.02). There was also no correlation between dedicated staff and TAT (Pearson’s correlation for all labs = −0.14; non-US labs = 0.009). There was a negative correlation for US laboratories with decreased TAT associated with increased numbers of staff and increased testing volume, but these were based on only three US laboratories (Pearson’s correlation US labs = −0.72 and −0.76, respectively).

Laboratories were asked whether there were national or regional guidelines to determine when to order a test and which genes to include on clinical tests for hereditary cancer syndromes (Supplemental Table 1 and 2). For the seven US laboratories that responded, three (43%) depended on the referring physician to determine appropriateness, two (29%) used National Comprehensive Cancer Network and/or payer/insurance guidelines and one (14%) used insurance carrier guidelines. For non-US laboratories, almost half (20 of 41 laboratories) depended on the ordering physician to determine the test being ordered and the other half (21 of 41) used specific guidelines. The specific guidelines for inclusion of a gene on a clinical panel for non-US laboratories varied: 12 of 41 laboratories (29%) had no country-specific guidelines, eight (20%) had defined genes that were covered by insurance, and 20 laboratories (49%) had regional or national level guidelines.

## Discussion

To our knowledge, this is the first international survey of laboratories performing clinical testing of *BRCA1/BRCA2* since the adaptation of MPS. MPS use for clinical testing has been predicted to introduce challenges in workflow, interpretation, and result reporting.^10^ By sampling the variety of technological approaches used by testing laboratories and highlighting similarities and differences, our goal is to provide information for testing laboratories and agencies providing guidelines for quality laboratory practices.

Across the world, MPS platforms were the preferred method for detection of non-rearrangement *BRCA1/BRCA2* sequence variants. All laboratories interrogated all coding *BRCA1/BRCA2* exons with varying lengths of introns sequenced. Although many survey responses were similar between non-US and US laboratories, our survey revealed some key differences. Non-US laboratories were more likely to use MLPA as one modality for large rearrangement detection (86%) compared to US laboratories (50%) who were more likely to use aCGH (75%) and MPS (88%). Most US and non-US laboratories utilized MPS as the prime modality for variant detection, but perhaps due to testing volume, more non-US laboratories used Illumina MiSeq (59%) instead of the higher capacity Illumina HiSeq used by most US laboratories (63%). US laboratories were much more likely to use in-house methods of classifying VUS (63%) compared to non-US laboratories (26%). Non-US laboratories were more likely to use country-specific or expert panel-specific *BRCA1/BRCA2* guidelines (21%); although three of eight US laboratories used ACMG or modified ACMG guidelines.^3^ US laboratories were more likely to share sequence results with public databases (88% reported to ClinVar) than non-US laboratories (55% reported to at least one public database). Importantly, over one third of non-US laboratories did not report variants to any databases or only reported to private/member only sites (9%). From a client point of view, US laboratories have a substantially shorter average TAT for *BRCA1/BRCA2* or small gene panels. This difference may be driven by competition between companies in the US, the distribution of commercial versus academic laboratories, and/or differences in insurance coverage versus national health care coverage for testing. Finally, responding US laboratories performed many more *BRCA1/BRCA2* tests (lowest reported 45,000) per year relative to non-US laboratories (range 1–2025), although only a small number of US laboratories answered this question. The vast difference in testing volume between US and non-US laboratories may be due in part to differences in the population size of responding countries or in numbers of patients referred for *BRCA1/BRCA2* testing. There was a strong correlation for US laboratories between TAT and number of *BRCA1/BRCA2* tests performed annually (Pearson’s correlation = −0.76); however there was no correlation for non-US laboratories (Pearson’s correlation = 0.02).

Although we aimed to do a global survey, a limited number of laboratories from Africa, South America, and Asia were sent direct survey links. As the survey was in English, language differences also may have contributed to a low response rate for laboratories in Asian, African, and South American countries. Additionally, as the survey was lengthy and some questions required additional research, some laboratories may not have participated. The US-response rate was low (8 of 27), but because some of the surveys to the non-US laboratories were sent by others through laboratory networks, we are unable to calculate response rate. This survey was not comprehensive and may be missing information on some key differences in practices, such as method of library preparation for MPS and rationale for choice of technologies utilized. Additionally, this survey was not designed to compare sensitivity and specificity of pathogenic variant detection or VUS calling which is an important consideration for establishing best practices. Finally, we did not collect information on the rationale behind the choice of technologies for each laboratory which could be useful for laboratories updating technologies for *BRCA1/2* variant identification.

**Fig. 5:**
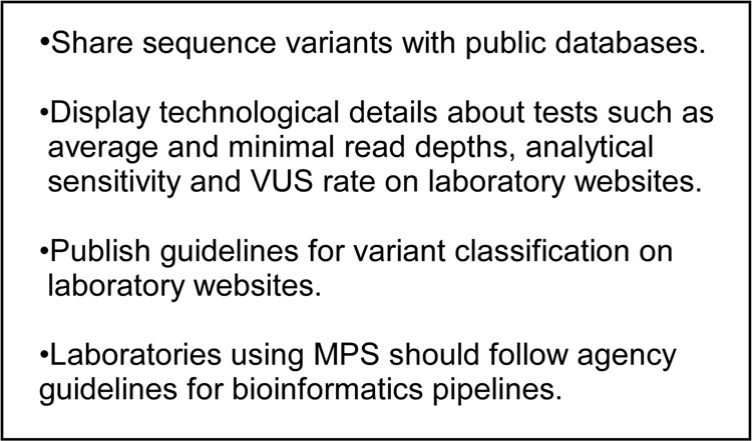
Points to consider. Points to consider which may improve client satisfaction and/or facilitate characterization of VUS are listed

In conclusion, this study shows that there are key similarities in technology used for *BRCA1*/*BRCA2* sequence and rearrangement analysis around the world. There was variation in laboratory-specific quality criteria for average and minimum numbers of read depths which could impact sensitivity. Laboratories reported VUS at varying rates, but also calculated rates of VUS differently. Based on these results and two recent studies, one showing a 2.6% diagnostic error rate for detection of pathogenic and significant variants using MPS approaches,^11^ and a second study suggesting that only two of seven MPS workflows could detect all 23 “challenging variants” in a blinded study^12^, we suggest points for consideration for laboratories performing or contemplating *BRCA* or panel testing (Fig. 5). These points for consideration (Fig. 5) are complemented by those from other groups, such as the Association for American Pathologist and the College of American Pathologist who have developed standards and guidelines for MPS Bioinformatics pipelines used in clinical tests.^13^ Of critical note, global data sharing of variants with sufficient data to allow multiple lines of evidence without duplication would facilitate variant reclassification and a central alert system to improve the quality of variant analysis and ensure more consistent management of high risk gene carriers as has been highlighted recently.^14,15^ Due to the diversity across laboratories, this study highlights the opportunity for international organizations to formulate guidelines for laboratories performing *BRCA1/BRCA2* genetic testing.

## Materials and methods

### Survey

Two online surveys were developed: one for non-US laboratories and one for US laboratories that included questions specific to the US (Supplementary Notes 1 and 2). Each survey had four sections: 1: Testing Laboratory Information, 2: Multigene Hereditary Cancer Panels, 3: Variant Assessment; and 4: Staffing, Billing and Other Client-Support Related Questions. Surveys were developed using Qualtrics Survey Software (Qualtrics, Provo, UT and Seattle, WA).

### Distribution of survey

Initial links to surveys were sent on 1 December 2016. Contact names and potential testing laboratories were identified through the BIC Steering Committee contacts, Google searches and mass e-mails sent from coordinators of the European Molecular Genetics Quality Network and United Kingdom National External Quality Assessment Service for Molecular Genetics to all genetics testing laboratories in their networks. Twenty-seven US laboratories were sent the survey. Survey links were distributed over a 4-month period. The international survey closed to new respondents on 29 March 2017 and the US survey closed to new respondents on 9 May 2017. For the non-US laboratories, 78 laboratories answered some questions, and 48 of those fully completed the survey, although some questions were not applicable to all laboratories (Supplementary Table 1). Eight US laboratories responded to the survey (Supplementary Table 2). The number of laboratories answering each question is included for each data point as the denominator varies.

### Data analysis

Summary data and frequencies were tallied in Qualtrics. As some questions had “click all that apply” answers, the percentage and number of laboratories responding are both reported. When a range of values was reported (e.g., 500–1000 read depth), the mid-point was used in the calculation (e.g., 750 reads). When a greater than or less than symbol was included (e.g., >100), the numerical value was used in the calculation. Answers related to time were converted to weeks.

### Map generation

The geographical locations of the participating laboratories were mapped by plotting the latitude and longitude markers obtained from the IP address of the individual completing the survey using ZeeMaps (https://www.zeemaps.com).

### Data availability

The authors declare that all of the data supporting the findings of this study are available within the paper and its supplementary information files.

## Electronic supplementary material

Supplementary Information Summary

Supplemental Note 1

Supplemental Note 2

Supplemental Table 1

Supplemental Table 2
