# Supplementary material for: Clinical testing of BRCA1 and BRCA2: a worldwide snapshot of technological practices
Source: NPJ Genom Med. 2018 Feb 15;3:7. doi: 10.1038/s41525-018-0046-7 (PMC5814433; doi:10.1038/s41525-018-0046-7)
Supplement: Supplementary file 1 — Supplementary Information Summary [file 41525_2018_46_MOESM1_ESM.docx]

**Supplementary Information Summary:**

SI File 1: Non-US survey questions

SI File 2: US survey questions

SI File 3: Supplementary Table 1: Survey questions and responses

SI File 4: Supplementary Table 2: Survey questions and responses
