# Supplementary material for: Clinical testing of BRCA1 and BRCA2: a worldwide snapshot of technological practices
Source: NPJ Genom Med. 2018 Feb 15;3:7. doi: 10.1038/s41525-018-0046-7 (PMC5814433; doi:10.1038/s41525-018-0046-7)
Supplement: Supplementary file 3 — Supplemental Note 2 [file 41525_2018_46_MOESM3_ESM.pdf]

US-Based Laboratory Technology Survey

Q59 The Breast Information Core (BIC) Steering Committee, in collaboration with Andrea Forman, MS, LCGC, of Fox Chase Cancer Center, is conducting a survey of laboratories that currently offer clinical testing for BRCA1 and BRCA2 and multigene hereditary cancer risk panels. Similar information was collected from several labs beginning in 2013 and data was recently presented at the 6th International Symposium on Hereditary Breast and Ovarian Cancer in Montreal. We recognize that additional laboratories now offer BRCA1/2 and/or multigene cancer risk panels and other labs have adjusted their technical specifications and resources since this information was originally requested. The goal of the survey is to obtain updated, side by side comparisons of the technology used for testing, what sequences are being evaluated, and additional information that is useful for clinicians when making decisions about which genetic testing laboratories best suit their needs and the needs of their patients.   We are asking a laboratory scientist or representative clinical geneticist from your laboratory to complete the information below. We appreciate that processes differ between labs both nationally and internationally, so we ask you to answer the questions to the best of your abilities reflecting the practices in your laboratory. We estimate that it will take 20-30 minutes to complete. We have attached a pdf of the survey for your review in the accompanying e-mail so you know what types of questions will be asked and what information you will need at hand in order to help facilitate completion of the online survey. The survey cannot be saved and accessed later, so we ask that it be completed at one time. This information will be made publically available.   We would appreciate completion of the survey by 12/30/2016. If you prefer not to participate, please let us know.   Thank you for your support. If you have any questions or concerns, please contact Andrea Forman, MS, LCGC at andrea.forman@fccc.edu or 215-728-7049.

Q61 Section 1: Testing Laboratory Information

Q68 Does your laboratory perform clinical genetic testing of BRCA1/BRCA2 and/or other genes associated with hereditary breast cancer?
- ❍ Yes (1)
- ❍ No (2)

If No Is Selected, Then Skip To End of Survey

Q1 Enter your Laboratory/Company Name
- ❑ Click to write Choice 1 (1) ____________________
- ❑ Opt Out: If you would prefer that your laboratory name remain unspecified in publications, please check here. All participating laboratory names will be made available to genetic counselors and other clinicians who reference this data for clinical care. (2)

Q2 Enter your location (City/Country)
- ❍ Click to write Choice 1 (1) ____________________

Q3 Name of Respondent/Contact Person
- ❍ Click to write Choice 1 (1) ____________________

Q60 E-mail of contact person
- ❍ Click to write Choice 1 (1) ____________________

Q69 CLIA Licence #
- ❍ Click to write Choice 1 (1) ____________________

Q70 CAP Accrediation #
- ❍ Click to write Choice 1 (1) ____________________

Q71 ISO-9000 Certification #
- ❍ Click to write Choice 1 (1) ____________________

Q72 Is your Laboratory Director boarded in genetics?
- ❍ Yes (1)
- ❍ No (2)
- ❍ Other (3) ____________________

Q62 Section 2: Multigene Hereditary Cancer Panels Please answer the following questions in regards to clinical testing with multigene cancer panels. Some questions are specific to testing of the BRCA1/2 genes only. If your lab only offers single gene (e.g. BRCA1/BRCA2) testing please answer accordingly.

Q4 What is the platform currently used by your laboratory for DNA sequencing of hereditary cancer risk genes? (Select all that apply).

- ❑ Illumina MiSeq (1)
- ❑ Illumina HiSeq (2)
- ❑ Ion Torent (3)
- ❑ NextSeq (4)
- ❑ Sanger Sequencing (5)
- ❑ Other (6) ____________________

Q5 What is the platform currently used by your laboratory for deletion/duplication analysis, when applicable, of hereditary cancer risk genes? (Select all that apply)

- ❑ MLPA (1)
- ❑ aCGH (2)
- ❑ Next-generation sequencing platform (3)
- ❑ Deletion/duplication analysis is not performed for any genes (4)
- ❑ Software analysis (e.g. CODEX) (5)
- ❑ Other (6) ____________________

Q6 Which genes are not analyzed for deletion/duplication?

- ❍ We do not perform deletion/duplication analysis for any genes (1)
- ❍ Deletion/duplication analysis is performed for every gene we assess (2)
- ❍ The following genes do not have deletion/duplication analysis performed (3) ____________________

Q7 Are all coding exons covered for genes in your hereditary cancer multigene panels? (Note exceptions in next question).

- ❍ Yes (1)
- ❍ No (list genes that are not covered in next question) (2)
- ❍ Other (3) ____________________

If Other Is Selected, Then Skip To Please describe any exons (and their ...If Yes Is Selected, Then Skip To Are the following promoter regions or...If No (list genes that are not... Is Selected, Then Skip To Please describe any exons (and their ...

Q8 Please describe any exons (and their affected gene) not covered in your multigene panels (e.g. only 1100delC assessed in CHEK2, no analysis of exons 12-15 in PMS2, single variant analysis of GREM1).

- ❍ Description (1) ____________________

Q9 Are the following promoter regions or specialty results included when the applicable gene is part of the panel? (Check all that apply).

- ❑ PTEN promoter (1)
- ❑ MLH1 promoter (2)
- ❑ MSH2 promoter (3)
- ❑ MSH2 Boland inversion (4)
- ❑ Sequencing of exons 12-15 in PMS2 (5)
- ❑ Other (6) ____________________

Q10 Are full intronic regions of any genes analyzed?

- ❍ Yes (1)
- ❍ No (2)

If No Is Selected, Then Skip To Are identified variants confirmed bef...

Q11 Which genes include full intron analysis?

- ❍ List (1) ____________________

Q12 Are identified variants confirmed using another method (or the same method) before reporting?

- ❍ Yes. Only pathogenic/likely pathogenic variants are confirmed (1)
- ❍ Yes. Both unclear and pathogenic/likely pathogenic variants are confirmed. (2)
- ❍ No (3)
- ❍ Other (4) ____________________

Q13 What methods/platforms are used to confirm identified genetic variants? (Check all that apply).

- ❑ Repeat sample analysis with original technology (1)
- ❑ Sanger Sequencing (2)
- ❑ MLPA (for deletion/duplication variants) (3)
- ❑ Base substitutions are not reexamined (4)
- ❑ Only frameshift mutations are examined (5)
- ❑ None (6)
- ❑ Other (7) ____________________

Q14 How many base pairs of intronic regions are typically assessed?

- ❍ up to 5 bp (1)
- ❍ 6-10 bp (2)
- ❍ 11-20 bp (3)
- ❍ All previously established clinically significant intronic variants (4)
- ❍ Other (5) ____________________

Q15 What regulatory regions are included in analysis of BRCA1 and BRCA2? (check all that apply)

- ❑ Promoters (1)
- ❑ Enhancers (2)
- ❑ Introns (3)
- ❑ 3'UTR (4)
- ❑ 5'UTR (5)
- ❑ None of the above (6)
- ❑ Other (7) ____________________

Q16 What is your analytic sensitivity of BRCA1/2?

- ❍ Describe (1) ____________________
- ❍ Unknown (2)

Q17 How many reference samples were used to determine BRCA1/2 analytic sensitivity?

- ❍ Number (1) ____________________
- ❍ Unknown (2)

Q18 What is your percentage of variants of uncertain significance (VUS) in BRCA1/2?

- ❍ Percentage (1) ____________________
- ❍ Not calculated (2)

Q19 How is the VUS rate calculated?

- ❍ Description (1) ____________________
- ❍ Not calculated (2)

Q20 For next-generation sequencing technology, what is your average depth of base pair reads across all genes?

- ❍ Average number reads (1) ____________________
- ❍ Not applicable (NGS not performed) (2)

Q21 What is your minimum depth of base pair reads needed to meet your quality criteria?

- ❍ Minimum (1) ____________________
- ❍ Not applicable (NGS not performed) (2)

Q22 For next-generation sequencing technology, what is your average depth of base pair reads for BRCA1/2?

- ❑ Average Number reads BRCA1 (1) ____________________
- ❑ Average number reads BRCA2 (2) ____________________
- ❑ Not applicable (NGS not performed) (3)

Q23 For BRCA1/2 what is your minimum depth of base pair reads?
- ❑ Minimum depth BRCA1 (1) ____________________
- ❑ Minimum depth BRCA2 (2) ____________________
- ❑ Not applicable (NGS not performed) (3)

Q24 How are "low read" regions or gaps in sequencing data evaluated? (Check all that apply)
- ❑ Repeat entire assay (1)
- ❑ Repeat assay around affected region (2)
- ❑ Sanger sequence affected region (3)
- ❑ No additional actions taken (4)
- ❑ Other (5) ____________________

Q25 What is the analytic sensitivity of your multi-gene panels?
- ❍ Analytic sensitivity (1) ____________________
- ❍ Do not perform multi-gene panels (2)

Q26 What is the sensitivity and false discovery rate (FDR)/positive predictive value (PPV) for single nucleotide variants?
- ❍ Sensitivity, FDR/PPV (1) ____________________

Q27 What is the sensitivity and FDR/PPV for indels?
- ❍ Sensitivity, FDR/PPV (1) ____________________

Q28 What size indels can be reliably detected?
- ❍ Size (1) ____________________
- ❍ Depends on gene (please describe) (2) ____________________

Q29 Does your laboratory offer variant-specific testing for familial pathogenic variants found in multigene panels?
- ❍ Yes (1)
- ❍ No (2)
- ❍ Other (3) ____________________

Q30 In September 2016, what was your average turnaround time for tests of less than 10 genes?
- ❍ Turnaround time (1) ____________________

Q31 In September 2016, what was your average turnaround time for single syndrome tests, e.g. BRCA1/2?
- ❍ Turnaround time (1) ____________________

Q32 In September 2016, what was your average turnaround time for a multigene panel of more than 10 genes?
❍ Turnaround time (1) ____________________

Q63 Section 3: Variant AssessmentPlease help us understand more about your variant analysis process as well as follow-up processes

Q34 Is variant interpretation performed by in-house staff?
❍ Yes (1)
❍ No (If no, where is it performed) (2) ____________________
❍ Other (3) ____________________

Q35 What variant interpretation guidelines do you follow?
❍ ACMG (1)
❍ In-house (describe) (2) ____________________
❍ Other (3) ____________________
❍ None (4)

Q36 Please describe your variant analysis process. Feel free to include a link if a detailed summary is available on your website. Please specify if there is a different process for BRCA1/2 analysis compared to a multigene panel.
❍ Description of process: (1) ____________________

Q37 How often does your laboratory re-assess previous variant results?
❍ Less than a year (1) ____________________
❍ Between 1-3 years (2)
❍ Greater than 3 years (3)
❍ Re-assessment is done on an Ad hoc basis (4)
❍ Not applicable. Our laboratory does not re-assess previous variant classification (5)

Q38 Is the ordering provider contacted when an unclear variant is reclassified?
❍ Yes, but only if reclassified as clinically actionable (e.g. pathogenic or likely pathogenic) (1)
❍ Yes, whether downgraded to benign/likely benign or upgraded to pathogenic/likely pathogenic (2)
❍ Providers are not automatically contacted (3)
❍ Reclassifications are shared only at the request of the ordering provider (4)
❍ Other (5) ____________________

Q39 Who is involved with the variant classification process? (Check all that apply)
❑ Board Certified Molecular Geneticist (1)
❑ Board Certified Medical Geneticist (2)
❑ Genetic Counselor (3)
❑ Individuals with clinical genetics expertise on the specific genes being studied (4)
❑ Expert classification panel (Specify details on panel here) (5) ____________________
❑ Other (6) ____________________

Q40 Do you offer variant-specific testing for family members when a VUS is identified for segregation and/or research studies (i.e. family studies)?
❍ Yes (1)
❍ No (2)
❍ Other (3) ____________________
If Other Is Selected, Then Skip To Who is involved in report writing? (C...If Yes Is Selected, Then Skip To If you offer family studies for VUS, ...If No Is Selected, Then Skip To Who is involved in report writing? (C...

Q41 If you offer family studies for VUS, please describe the process, including the required medical information, cost to patient, and turnaround time.
❍ Description of family studies for VUS: (1) ____________________

Q42 Who is involved in report writing? (Check all that apply)
❑ Board Certified Molecular Geneticist (1)
❑ Board Certified Medical Geneticist (2)
❑ Genetic Counselor (3)
❑ Individuals with clinical genetics expertise on the specific genes being studied (4)
❑ Other (5) ____________________

Q43 Which public variant databases do you contribute data? (Check all that apply).
❑ ClinVar (1)
❑ InSight (2)
❑ Global Alliance (3)
❑ LOVD (4)
❑ BIC (5)
❑ Other (Specify) (6) ____________________
❑ Our laboratory does not contribute variant data to public databases (7) ____________________

Q44 Do you respond to inquires about variants found in other laboratories?
❍ Yes (1)
❍ No (2)
❍ Other (3) ____________________

Q45 Are you willing to share a sample report showing how VUS results are reported to clinicians?

- Yes (please e-mail Andrea.Forman@fccc.edu) (1)
- No (2)
- Other (3) ____________________

Q64 Section 4: Staffing, Billing and Other QuestionsPlease help us better understand the staffing as well as billing and insurance processes for your lab/company. Some questions may not apply in countries with national health systems.

Q46 How many index cases for hereditary cancer risk were analyzed in your laboratory between October 2015-September 2016?

- Index case number: (1) ____________________

Q47 How many dedicated staff (full time equivalents) work at your company?

- Staff size: (1) ____________________

Q48 Does your lab/company have board certified genetic counselors on staff?

- Yes (1)
- No (2)
- Other (3) ____________________

If Yes Is Selected, Then Skip To How many genetic counselors or simila...If No Is Selected, Then Skip To Are there any guidelines (national or...If Other Is Selected, Then Skip To How many genetic counselors or simila...If Yes Is Selected, Then Skip To How many genetic counselors or simila...

Q48 How many genetic counselors or similar positions do you have on staff?

- Number: (1) ____________________

Q49 Describe the various roles genetic counselor (or equivalent positions) have within the lab/company. (Check all that apply).

- ❑ Direct patient education (e.g. telephone genetic counseling or availability to answer questions (1)
- ❑ Direct medical provider education (e.g. face to face or telephone contact for clinical questions) (2)
- ❑ Educational lectures for the lay public (3)
- ❑ Educational lectures for healthcare providers (4)
- ❑ Variant assessment and research (5)
- ❑ Writing result resports (6)
- ❑ Development of patient educational materials (7)
- ❑ Development of marketing materials (8)
- ❑ Coordination/writing of research publications (9)
- ❑ Other (10) ____________________

Q50 Are there any guidelines (national, regional or state) used to determine whether or not an ordered test is appropriate?
- No. We depend on the ordering clinician to determine appropriateness of testing (1)
- Yes, we use the following guideline(s): (2) ____________________

Q51 Are you able to bill private health insurance for tests (assuming patients meet appropriate criteria)?
- Yes (1)
- Testing is billed through national health system (2)
- No. Patient is self-pay (3)
- Not applicable (4)
- Other (5) ____________________

Q73 Are you able to bill Medicare for tests (assuming patients meet appropriate criteria)?
- Yes (1)
- No (2)
- Not applicable (e.g. all patients are self-pay) (3)
- Other (4) ____________________

Q74 Are you able to bill Medicaid (state or managed) for tests (assuming patients meet appropriate criteria)?
- Yes (1)
- No (2)
- Not applicable (e.g. all patients are self-pay) (3)
- Other (4) ____________________

Q52 Do you provide pre-verification services for insurance coverage?
- Yes, once a sample is received (1)
- Yes, once a sample is received OR if requested prior to sample submission (2)
- No (3)
- Not applicable (4)
- Other (5)

Q53 Do you offer financial assistance options?
- Yes [Please describe financial assistance options including income and documentation requirements (e.g. (1) ____________________
- No (2)
- Not applicable (3)
- Other (4)

Q54 Do you offer genetic testing of DNA from buccal samples?
- Yes (1)
- No (2)
- Other (3) ____________________

Q55 Do you offer genetic testing on skin fibroblasts?
- Yes (1)
- No (2)
- Other (3) ____________________

If Other Is Selected, Then Skip To Are you able to culture skin fibrobla...If Yes Is Selected, Then Skip To Are you able to culture skin fibrobla...If No Is Selected, Then Skip To Do you offer genetic testing internat...If Yes Is Selected, Then Skip To Are you able to culture skin fibrobla...

Q56 Are you able to culture skin fibroblasts or lymphoblastoid cell lines within your lab?
- Yes (1)
- No (2)
- Other (3) ____________________

Q57 Do you offer genetic testing internationally?
- Yes (1)
- No (2)
- Other (3) ____________________

If Yes Is Selected, Then Skip To If international testing is available...If Yes Is Selected, Then Skip To If international testing is available...If Other Is Selected, Then Skip To End of SurveyIf No Is Selected, Then Skip To End of Survey

Q58 If international testing is available, do you cover shipping costs to submit samples?
- Yes (1)
- No (2)
- Other (3) ____________________
